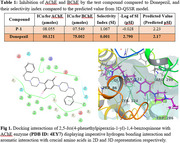# 2,5‐*Bis*(4‐phenethylpiperazin‐1‐yl)‐1,4‐benzoquinone: Harnessing AChE Selectivity for Alzheimer's Disease Therapy.

**DOI:** 10.1002/alz70859_100743

**Published:** 2025-12-25

**Authors:** Pragati Silakari, Aditi Yadav, Poonam Piplani

**Affiliations:** ^1^ Chitkara College of Pharmacy, Chitkara University, Rajpura, Punjab India; ^2^ Panjab University, Chandigarh, Chandigarh India

## Abstract

**Background:**

The present study recounts the potency of the novel synthesized **2,5‐*Bis*(4‐phenethylpiperazin‐1‐yl)‐1,4‐benzoquinone (P‐1)** as a potent molecule selectively targeting AChE along with the antioxidant action that helps to manage cognitive decline in Alzheimer’s disease.

**Method:**

Synthesis of novel **2,5‐*Bis*(4‐phenethylpiperazin‐1‐yl)‐1,4‐benzoquinone** was performed by utilizing appropriate synthetic procedures followed by characterization using various spectral and elemental techniques. The techniques of TLC, Melting Point Determination and Elemental analysis were employed to establish the purity of this synthetic analogue. This was followed by the use of mice homogenate as the source of enzyme to evaluate of its selective in vitro acetylcholinesterase (AChE) and butyrylcholinesterase (BChE) inhibitory potential at different concentrations. Further evaluation of its antioxidant potential was performed using DPHH radical scavenging and hydrogen peroxide radical scavenging protocols at five different concentrations followed by study against behavioural alterations at a dose of 0.5 mg/kg with reference to the standard drug donepezil using step down passive avoidance and escape learning protocol. To induce dementia in animal model a dose of 2.0 mg/kg scopolamine was used. The mice brain homogenate was used as the source of enzyme to perform ex vivo AChE inhibition. Also, to determine the role of the synthesized molecule, on the oxidative damage induced by scopolamine, biochemical estimation of the markers of oxidative stress which include lipid peroxidation, glutathione, catalase and superoxide dismutase has also been carried out.

**Result:**

A synergistic effect of two structural components on the inhibitory activity against AChE has been anticipated. The synthetic analogue possessed selective AChE activity (**IC_50_ Value: 8.055 µmoles**), an appreciable value of selectivity index (**1.067**) and significant antioxidant potential (Table 1). The ex vivo AChE inhibition assays complied with the behavioural studies data along with the docking results showing important interactions with AChE protein (Figure 1)

**Conclusion:**

The results showed memory restorative effect of the synthesized compound on scopolamine‐induced memory impairment most likely being related to mediation of synaptic transmission in cholinergic system and their effective antioxidant activity. Hence, the results were appreciable and provided a promising lead which can further be explored for the development of selective AChE inhibitors.